# Heterospecific Fear and Avoidance Behaviour in Domestic Horses (*Equus caballus*)

**DOI:** 10.3390/ani11113081

**Published:** 2021-10-28

**Authors:** Anna Wiśniewska, Iwona Janczarek, Izabela Wilk, Ewelina Tkaczyk, Martyna Mierzicka, Christina R. Stanley, Aleksandra Górecka-Bruzda

**Affiliations:** 1 Department of Horse Breeding and Use, Faculty of Animal Sciences and Bioeconomy, University of Life Sciences in Lublin, 20-950 Lublin, Poland; anna.wisniewska@up.lublin.pl (A.W.); iwona.janczarek@up.lublin.pl (I.J.); izabela.wilk@up.lublin.pl (I.W.); ewelina.tkaczyk@up.lublin.pl (E.T.); khiuk@up.lublin.pl (M.M.); 2 Animal Behaviour & Welfare Research Group, Department of Biological Sciences, University of Chester, Chester CH1 4BJ, UK; christina.stanley@chester.ac.uk; 3 Department of Animal Behaviour and Welfare, Institute of Genetics and Animal Biotechnology, Polish Academy of Sciences, 05-552 Magdalenka, Poland

**Keywords:** fear, horse, cattle, avoidance, heterospecific interaction, sex differences

## Abstract

**Simple Summary:**

Horses lacking exposure to cattle often show a fearful response when confronted with cows. In controlled conditions, we tested the responses towards two cows and a novel moving object in twenty horses in arena and hand-leading tests. The horses avoided the proximity of all stimuli, but of one of the cows the most. However, both cows provoked a stronger cardiac response than an inanimate object. We hypothesise that this response may be due to both neophobia and heterospecific interactions.

**Abstract:**

Ridden horses have been reported to be fearful of cows. We tested whether cows could provoke behavioural and cardiac fear responses in horses, and whether these responses differ in magnitude to those shown to other potential dangers. Twenty horses were exposed to cow, a mobile object or no object. The time spent at different distances from the stimulus was measured. In a separate test, heart rate (HR), root mean square of successive differences between heartbeats (RMSSD) and the horses’ perceived fear were assessed at various distances from the stimuli. The horses avoided the area nearest to all stimuli. During hand-leading, the cow elicited the highest HR and lowest RMSSD. Led horses’ responses to the cow and box were rated as more fearful as the distance to the stimulus decreased. Mares had a higher HR than geldings across all tests. HR positively correlated with the fearfulness rating at the furthest distance from the cow and box, and RMSSD negatively correlated with this rating in cow and control conditions. Our results show that these horses’ avoidance response to cows was similar or higher to that shown towards a novel moving object, demonstrating that potentially, both neophobia and heterospecific communication play a role in this reaction.

## 1. Introduction

Historically, bovines and working equines were often kept and grazed together in extensive rural systems, meaning they were habituated to each other’s presence. Several projects reintroducing horses and cattle to pre-domestication, semi-natural conditions have resulted in successful, peaceful coexistence between free-roaming horses and cattle, each of them forming stable species-specific groups [1]. Despite an apparent tolerance between horses and cows when co-grazing, there have been reports from local riders to the authors (personal communications) of leisure horses showing a fearful response to cows. At present, in many countries, both species are kept in separate facilities so they rarely, or never, meet in pastures or yards. In particular, when horses are housed for riding in suburban areas, mostly being exercised in controlled environments such as indoor or outdoor arenas, they do not experience contact with other ungulates of a similar size. When suddenly exposed to a cow during riding outside the equestrian centre, many horses thus respond with fear and avoidance behaviours.

Fearfulness varies in magnitude between individual horses. It can be defined as a “basic psychological characteristic of the individual that predisposes it to perceive and react in a similar manner to a wide range of potentially frightening events” [2]. Fear in ridden horses is often associated with a sudden stop, alarmed posture, intense staring at the novel object and, when the alarm level is high enough, it can provoke a flight response [3]. A spooked horse can be hard to control by its rider; considering the speed of escape, accidents during horse riding can put a rider in significant danger [4]. As a result of its high relevance to riders and handlers of horses, fear has been the object of several scientific studies e.g., [5]. During ridden work, horses are frequently asked to accept events and objects that they would normally avoid [6]. Specifically, the readiness to react with alarm-associated behaviour in response to unfamiliar, unexpected objects is often used to quantify fearfulness in horses during standardized tests [7]. The objects used are inanimate, often mobile, objects and have previously included, for example, rotating balloons [8], boxes with “hands and legs” and a novel surface [9], a lifted up black plastic bag [10], a plastic container filled with wooden sticks [11] or a children’s plastic playset [12] and many other. For horses, it is often not the novelty of a stimulus but the suddenness of it being introduced that is perceived as more fear-eliciting [2,12]. Therefore, mobile objects, including cars or other machines, often provoke alertness and avoidance in horses (AGB, practical observations). However, to our knowledge, the fear response to an unfamiliar, similar-sized animal has not previously been tested in horses in standardised conditions.

The aim of the present study was to investigate whether domestic horses show specific fearfulness towards cows. We tested the reaction of horses to the presence of a cow compared with a novel mobile object to elucidate whether cows can provoke behavioural and cardiac fear responses in horses, and whether these responses differ in magnitude to those shown to other potential dangers [13]. Considering that horses and ungulates such as cows or sheep commonly coexist peacefully in rural settings, and also that in evolutionary history, wild equids are likely to have formed mixed species herds with other ungulate species as protection against predators, we predict that horses are likely to be less fearful of cows than of mobile novel objects.

To assess the fear response, behavioural reactions indicative of alarm and avoidance and physiological measures of stress (heart rate, HR, and the root mean square of successive differences between heartbeats, RMSSD) were recorded, as both are confirmed indicators of the stress and flightiness e.g., [14,15,16]. Finally, we investigated whether human evaluation of the magnitude of a horse’s fearfulness toward an unfamiliar object correlated with physiological measures of fear; it was reported that an experienced horse handler can reliably assess fear levels [8,17]; therefore, this could have implications for rider and handler safety.

## 2. Materials and Methods

### 2.1. Ethical Note

The study involved the analysis of observations of animals and mimicked normal conditions at an equestrian club. No invasive experimentation was performed in view of European directive 2010/63/EU and the Polish laws related to ethics in animal experimentation. The horses belonged to the University of Life Sciences in Lublin, Poland, and were maintained in a riding centre under the care of one of the authors (I.J.) who monitored their welfare as assigned by the university. The procedures took place in a familiar environment and did not cause them any pain, suffering, or damage. The manager of the facility allowed the experiment to be performed since common turnout of horses and cattle was intended in future on pasture. The habituation of the two species of animals to each other became a standard procedure at the time of the experiment. The moving box was in line with the manager’s plan to habituate the horses to new objects by desensitization. In this way, horses were trained to travel outside the facility to be safer for riders.

### 2.2. Animals

Twenty riding school warmblood horses, 13 geldings and 7 mares, with a mean age of 13 ± 5.2 years, were used in the study. They were housed in individual box stables with straw bedding, fed with commercial pellets and hay, and a salt block and water were available *ad libitum*. The horses were grazed in groups in paddocks for six hours daily. They were ridden for on average of seven hours per week, with an approximately equal workload in terms of physical exercise. During the study period, all horses were clinically healthy. All horses had previous incidental experience in terms of visual contact with two cows inhabiting a separate building within the riding centre (located about 200 m from stables) for five months prior to the onset of the study, but not when (infrequently) out hacking. No close contact or common pasturing was practiced between horses and cows at this centre.

Two tests were carried out between June and September 2020 in the equestrian centre where the animals were stabled: an arena test and a hand-leading test. During the tests, the ambient temperature ranged from 19 to 24 °C.

### 2.3. Arena Test

**Figure 1:**
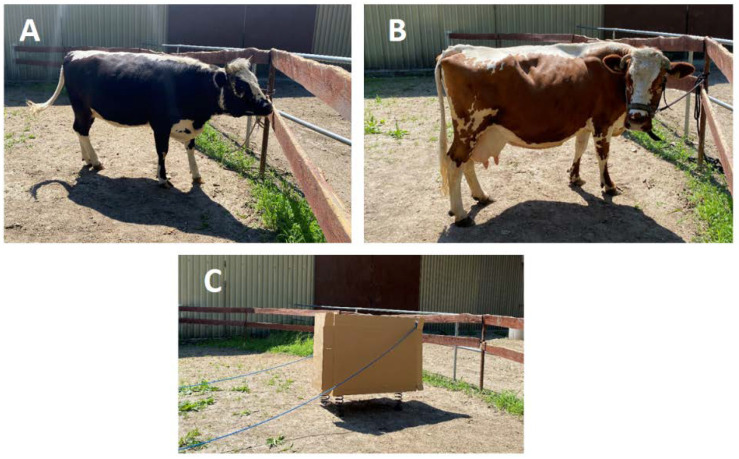
The stimuli used in the study: Cow1 (**A**), Cow2 (**B**) and the Box (**C**). The cows were tethered for the photo but free to move during the study. Two electric cables power the mechanism hidden in the cardboard box.

The horses were submitted to a test of their voluntary responses to freely-moving cows or a novel mobile box in the arena test. Two different cows were successively presented as stimuli to determine if each would elicit similar response in horses. During testing, care was taken for the horses not to be potentially distracted by humans, other horses or vehicles passing in close proximity. The uncovered rectangular testing arena (20 × 40 m), familiar to these horses, was divided into three sectors (marked by a line scratched on the ground); Sector1 was the nearest and Sector3 was farthest from the stimulus presentation arena (Figure 1A). On one long side of the testing arena was the wall of a building; on the other side, there was a paddock where a familiar companion horse (always the same individual) was placed during testing. The stimuli were presented on the short side of the testing arena (stimulus exposure arena, 20 × 10 m, Figure 1A). These were two cows of different sizes and colours (Cow1, height at withers 1.17 m, Polish Black White-Backed breed; Cow2, height at withers 1.23 m, Polish Red White-Backed breed, Figure 1A,B), and a novel cuboid cardboard box (1.2 × 1.8 × 0.6 m, Figure 1C), with a mechanism enabling automatic movements in four directions by approximately 15 cm in a standardized manner (Box). The arenas and paddocks were fenced to enable free contact between the animals, including tactile contact. Water was provided to cows and horses *ad libitum*.

**Figure 2:**
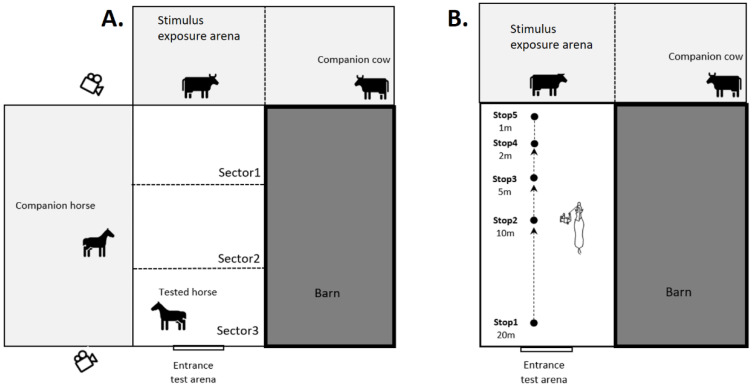
Schematic representation of (**A**). Arena test and (**B**). Hand-leading test.

At the start of a testing session, a horse was brought into the test arena and allowed to move freely around the arena for 30 min, while a companion horse was present in an adjoining paddock (Control Stay). After 30 min of the Control Stay condition, the stimulus (Cow1) was introduced to the neighbouring stimulus exposure arena and remained there for a further 30 min. The companion cow (Cow2) was in a separate enclosure adjoining the stimulus exposure arena, in the part not visible to the tested horse but visible to Cow1 (Figure 2A). After 30 min, Cow 1 was replaced by Cow2 for another 30 min. The order in which the two cows were presented was allocated randomly amongst the horses, with half experiencing Cow1 first and half experiencing Cow2 first. After one month, the test was repeated following the same protocol, but with a novel (Box), placed in the centre of the exposure arena, replacing the cow. The box was activated before the arrival of the horse and set to move in a random order, during the duration of the test phase (30 min). Therefore, each horse was exposed to 3 different stimuli: Cow1, Cow2 and the Box, preceded by the Control Stay condition, each of duration of 30 min. Two cameras (Sanyo Xacti VPC-WH1, Moriguchi, Japan) installed on two sides of the testing arena recorded the behaviour of the horse being tested. The horse’s behaviour was later scored by an experienced researcher (AW) in terms of the duration of the stay in the chosen sector and the frequency and total duration of various “alert” behaviours (Table 1) by viewing the recorded footage.

### 2.4. Hand-Leading Test

Two months after the arena test with the Box, the same horses, equipped with a telemetry device (Polar RS800CX, Polar Oy, Finland) to which they were habituated during periodic standard health examination, were individually hand-led to the testing arena by a handler (always the same, experienced person). Care was taken that the horses were not exposed either to cows or to the box between these test phases. The overview of the hand-leading test is presented in Figure 2B. The horse was led into the testing arena and stopped for 10 s at five different positions (marked by a line scratched on the ground) in succession: 20 m (Stop1), 10 m (Stop2), 5 m (Stop3), 2 m (Stop4) and 1 m (Stop5) distances from the previous location of the stimuli (control lead). The handler did not use any voice command and led the horse in the manner to which they were accustomed, with only slight tension on the lead rope when encouraging the horse to approach the stimulus presentation arena. At each stop, the horse was held on a loose lead rope to enable its movement around the handler who was not allowed to interact with the horse. After one week, the hand-leading test was repeated, and half of the horses being asked to approach the cow (Cow1 or Cow2, whichever was used last in that individual horse’s arena test) and the other half approaching the Box (with the horses being allocated randomly to each of these two conditions). The same day, the groups were then swapped, and individuals were asked to approach the other stimulus (Cow or Box). During the test, the experimenter (AW) observed the horses from about 10 m, standing outside the fence, at the level of Stop2.

The intensity of perceived fearfulness of the horse at each stop was scored by the experimenter during testing by them marking a point on a visual analogue scale (VASf, see Table 1). VASf rating has been used in previous studies for the assessment of subjective feelings in humans e.g., [18,19] and, in equine veterinary science, to measure the magnitude of pain in the horse e.g., [20,21]. For each stop, the heart rate (HR) and the root mean square of successive differences between heartbeats (RMSSD) in 10-s period were later extracted from the monitor’s files.

The variables were analysed using Polar ProTrainer5 software. No filter level was applied since clear, almost artefact-free RR curves were recorded (in three files the % of artefacts were 0.4%; 0.7%; 1.3%) [22,23].

### 2.5. Statistical Analysis

All analyses were performed in the SAS statistical package (SAS 9.4., SAS Inst., Inc., Cary, NC, USA).

The occurrences of alert behaviours and their durations were extremely low (mean ALERT-occur: 2.90 ± 4.83, and ALERT-dur: 8.83 ± 32.6 s), so these variables were not further analysed. For time spent in sectors (TimeSector), the effect of the sector (Sector1, Sector2 and Sector3), the stimulus (Control Stay, Cow1, Cow2/cow, Box), the interaction of stimulus with the sector; the sex (gelding, mare) and age (below or above 10 years old) were assessed by applying generalised mixed models (GLIMMIX), assuming a binomial distribution for proportions of observed durations related to the total time of observations (for TimeSector). The HR did not follow normal distribution, and thus was log-transformed and Gaussian distribution was confirmed by a Kolmogorov–Smirnov test. For HR, RMSSD and VASf, the effect of the stop, the stimulus, the interaction of stimulus with the stop, the sex and age were assessed by applying GLIMMIX, assuming Gaussian distribution for HR and RMSSD and a Poisson distribution for VASf. All models included a random factor of animal ID and a Tukey adjustment accounting for repeated measures on the same animals. The ILINK option of SAS was used to compute the estimates and standard errors on the original scale of variables; for back-transformed values, confidence intervals were presented as a measure of variance.

To examine how well the assessment of fear is reflected by physiological measures, a Spearman’s correlation was carried out between VASf scale and either HR or RMSSD in the Control Lead condition, and when the Cow and Box was present in the hand-leading test. The analyses were performed for VASf and both cardiac variables at Stop1 (the farthest to the stimulus) and Stop5 (the nearest to the stimulus), as these two stops differed by the greatest distance.

## 3. Results

### 3.1. Arena Test

The effects of the stimuli, sectors, age and sex on TimeSector (time spent in each sector of the arena) are shown in Table 2. We did not find the age of the horse to significantly predict any of the studied variables.

The horses spent significantly different percentages of time in each sector according to the stimulus presented (Table 2).

Across all conditions, the horses significantly avoided Sector1 in comparison to Sector2 (*t* = 23.5, *p* < 0.01) and Sector3 (*t* = 142.4, *p* < 0.01), which was most preferred area to stay in (Sector2 vs. Sector3, *t* = 163, *p* < 0.01). All detailed comparisons are provided in Table 3.

Within all sectors, when comparing the times spent in the presence of different stimuli, the most avoided stimulus was Cow1, followed by Box, Cow2 and the Control Stay. The most aversive stimulus, Cow1, provoked the least time being spent in the sector nearest to the stimulus (Sector1) compared with Control Stay (*t* = 39.2, *p* < 0.01), Cow2 (*t* = 39.7, *p* < 0.01) or the Box (*t* = 24.2, *p* < 0.01). The Box was less aversive than Cow1 but provoked less time to be spent in Sector1 than did Cow2 (*t* = 16.0, *p* < 0.01), and the Control Stay (*t* = 15.5, *p* < 0.01). Both Cow2 and Control Stay were best tolerated by horses in Sector1 (*t* = 0.46, *p* = 1.00).

In the middle sector (Sector2), TimeSector was higher when horses were confronted with Cow1 as compared to the Box (*t* = 5.91, *p* < 0.01), Cow2 (*t* = 15.2, *p* < 0.01) and the Control Stay (*t* = 12.4, *p* < 0.01), while the Box provoked more time to be spent in Sector1 than Cow2 (*t* = 9.43, *p* < 0.01, and the Control Stay (*t* = 6.32, *p* < 0.01). Both Cow2 and Control Stay provoked the least avoidance of Sector1 (*t* = 3.12, *p* = 0.08).

In the sector farthest from the stimulus (Sector3), again Cow1 was responsible for horses choosing this sector for longer stays as compared to the Box (*t* = 8.73, *p* < 0.01), Cow2 (*t* = 23.0, *p* < 0.01) and the Control Stay (*t* = 25.1, *p* < 0.01). The Box provoked more time to be spent in Sector3 than both Cow2 (*t* = 16.4, *p* < 0.01) and the Control Stay (*t* = 8.73, *p* < 0.01), that did not differ in terms of TimeSector (*t* = 2.09, *p* = 0.63).

### 3.2. Hand-Leading Test

The effects of the stimuli, stop, sex and age on HR, RMSSD and VASf are shown in Table 4.

The HR and RMSSD results in the hand-leading test are presented in Table 5. The HR differed between stimuli and sexes (Table 4). Generally, the HR increased when the Cow was presented; this resulted in a higher HR than Control Lead (by 13.9 bpm, *t* = 13.6; *p* < 0.01) and the Box (by 14.9 bpm, *t* = 11.5; *p* < 0.01), while the HR in response to Control Lead and Box did not differ (*t* = −1.09; *p* = 0.52). Mares generally had a higher HR than geldings (by 6.7 bpm, *F* = 5.30, *p* = 0.02).

Similarly, RMSSD was affected by the stimulus and the sex of the horse (Table 4). The Cow evoked a lower RMSSD than Control Lead (by 28.4 msec, *F* = 12.0, *p* < 0.01) and the Box (39.7 msec, *F* = 14.0, *p* < 0.01), while the last two did not differ (*F* = 0.46, *p* = 0.67). Mares generally showed a lower RMSSD than geldings (by 28.7 msec, *F* = 6.48, *p* < 0.01).

**Figure 3:**
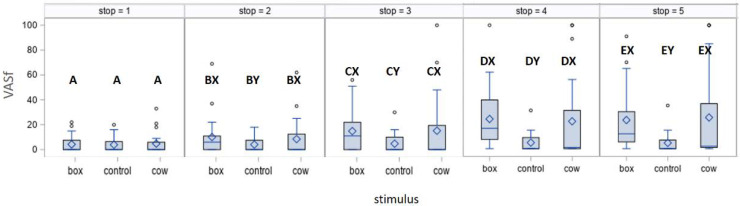
The assessment of fearfulness of horses (VASf) exposed to different stimuli at stops from the furthest (Stop1) to the nearest (Stop5). Scores with A–E letters differ significantly at *p* < 0.01 between stops; scores with X, Y letters differ significantly at *p* < 0.01 between stimuli; values with no letter do not differ to other values. Diamonds represent median values, borders of boxes occur at upper and lower quartiles and blue lines indicate maximum and minimum values. Circles show outliers.

The HR and RMSSD did not differ between stops (Supplementary Material, Figures S1 and S2), while the VASf did (Table 4). The fearfulness rating differed according to the stimulus. In human assessment, the Cow and the Box caused more fearfulness at each stop (except for Stop1) than the Control Lead (detailed comparisons in Figure 3). Except for the Control Lead, the VASf increased significantly between subsequent stops.

For all stops, VASf was significantly, but weakly, positively correlated with HR (*r_s_* (*n* = 20) = 0.22, *p* < 0.01) and negatively correlated with RMSSD (r_s_ (*n* = 20) = −0.24, *p* < 0.01). The correlations between the HR, RMSSD and VASf at the stop the farthest from the stimulus (Stop1) and the nearest to the stimulus (Stop5) during the hand-leading test are presented in Table 6. At the farthest Stop1, the subjective (VASf) and objective (HR and RMSSD) measures were correlated for four (five including the tendency for RMSSD in Box lead) from six possible correlations, showing a moderate agreement between the handler’s assessment of the behaviour and the physiological response of the horse during the lead when the cow and the box were present. At the nearest distance (Stop5), only the RMSSD during leading towards the Box correlated negatively with VASf (*p* = 0.05).

## 4. Discussion

The reaction of the studied horses to both animate and inanimate stimuli, i.e., their levels of alertness and the cardiac response, did not strongly differ from those in the control stay or lead, meaning both objects were quite well tolerated. However, the avoidance of the stimuli and changes in cardiac activity (increased sympathetic and decreased parasympathetic autonomic responses) could still be observed. Our results confirmed the hypothesis that in horses that are not habituated to cows, the response is similar, or even higher, in terms of fearfulness or avoidance, as to an inanimate novel moving object. In contrast to our expectations, cows were not better tolerated by horses than a novel, mobile object. Generally, the selection of the area further from stimuli observed in the arena test was indicative of higher avoidance of, specifically, one of the cows, while the cardiac activity in the hand-leading test showed that both cows provoked an avoidance response, which was higher than in box and control treatments. However, human assessment did not differentiate the between behaviour of horses approaching the different stimuli, with the researcher perceiving the behaviour of the horses when leading towards the cows and the box as equally fearful.

Our results confirm previous findings that horses, as prey animals whose initial reaction to potential threats is often flight, are sensitive to (potentially) all unknown frightening stimuli [2], and this propensity to react with fearfulness [4], as encoded in equine temperament [24], still exists despite years of domestication and selection against fearful behaviour [11]. Instinctive reactions to mobile objects, living or not, provoke avoidance in horses [25,26]. In a ridden or hand-leading situation, in particular, when horses are restrained, the restraint per se may provoke a stress response [27]. Moreover, the emotions of riders may be transferred to horses [28,29], which can affect the perception of objects as more frightening [30]. This fact can be explanative of similar perception of the two stimuli and the experimenter’s likelihood to expect a fear response when horses were led towards either stimulus.

In our tests, the examination of voluntary behaviour of horses when released in an open space of test arena showed that the horses typically did not approach the cow, except for one horse that accepted very close contact with the cow, including her licking the horse’s face. In the hand-leading test, all horses, except one individual, allowed the approach to the stimulus, although they were reluctant to approach them voluntarily, which confirm Christensen’s [31] results, that trained horses rather easily habituate to objects they previously avoided. Since horses, similar to other domestic animals such as dogs [32] or goats [33], seek human support when confronted with a challenging task [34], the presence of the handler could also in this case play a supportive role [35]. It seems then, that a cow is just another novel object to which the horses, when appropriately trained, can easily habituate.

In the hand-leading test, the researcher assessed the fearful response of horses to the cow and the box as being at similar levels, especially when approaching them to the closest point. This was particularly evident in the rating of behaviour during the Control Lead, where the handler did not expect any fearful reaction from horses, especially at Stop1. However, as shown by cardiac responses, the cows were perceived by horses as more frightening than another object, a mobile box. This result shows that even when alerted, horses are able to assess to some degree of level of the aversiveness of the object they are afraid of. As shown by Lansade et al. [24], some objects provoking fearful behaviour were no longer frightening after six months or longer, while the others caused still the same fearful response at each of the exposures over a two-year duration. This evidences that horses can individually assess and behave differently toward objects to which they are exposed. In particular, discrimination between individual cows, and a more fearful response to one of them, agree with previous reports that horses possess cognitive abilities that enable discrimination between conspecifics [36] or other species, such as humans [37]. These abilities are evident considering that horses are social animals, where recognition of familiar or unfamiliar individuals is crucial for the formation of groups and for maintaining social hierarchies [38].

Despite a researcher’s assessment of increasing fearfulness in horses as they approached stimuli, the HR of horses did not significantly rise when approaching the stimuli. This result was probably related to the low variability in the heart rate, which remained at the same level from the farthest to the nearest stop, indicating that the horses had already ‘physiologically’ reacted to the stimulus, and specifically to the cows, from the distance of 20 m. Since horse vision is characterised by low acuity [39,40], our results are explanative of the typical instantaneousness of reaction of the horses that flee long before the inspection of potentially dangerous object.

Although we showed here that in the studied horse population, cows provoked higher avoidance responses than a similar-sized inanimate mobile object, our study does not explain why this was so. Within this study, we also cannot explain why Cow1 was more frightening for the horses. It is possible that the behaviour of cows, rather than that of the box, was more unpredictable for horses. It has been shown in the previous studies [41,42] that humans and other species can discriminate and specifically react to biological motion. It is possible that the difference in the movement characteristics between the inanimate object (Box) and the cows was perceived by the horses and, consequently, their reactions were different. Despite both cows being presented in a standardized way and that they were mainly standing ruminating, we could not exclude some heterospecific communication, such as by odour, possible vocalisation and movement that could be attributed only to living organisms [43]. However, considering that voluntary mixed-species grouping and communication, for instance as an anti-predatory strategy [44], are practiced by African ungulates including equids (plains zebras, *Equus quagga*), the current practice of separating horses and cows is likely to best explain the (initial) alert and avoidance behaviour. In the long-term, as observed by Sablik et al. [45], interspecific communication during common pasturing becomes evident: alarmed horses were mostly ignored by the cattle herd, but alarmed cattle were followed by horses. We can therefore hypothesise that not only neophobia is involved in the avoidance of cows. Our results could be further enhanced by studies on familiarity and habituation to cows and other animals, which could shed more light on interspecific communication between cows and horses.

Finally, our study indicates a stronger physiological (cardiac) response in mares during the hand-leading test. More cautious behaviour seems justified in females which, by nature, are often both pregnant and with a foal at foot. The differences in reactivity between horses of different sexes, including fearfulness, and the relative welfare of mares and geldings, are currently under scientific debate [46,47]. The confirmation of the existence of sex differences in equine fear behaviour depends on the approach, type of animal and measures used, with some studies confirming sex differences e.g., [47] and others failing to do so [16]. Sex differences in fearful behaviour have been confirmed in a range of animals, including farm e.g., [48,49] and laboratory e.g., [50] animals, meaning this should be considered when studying and responsibly using horses.

## 5. Conclusions

In this study, we found cows to be the objects that horses preferred to avoid. Although the two species studied can be successfully pastured together, it seems that in the restricted conditions of a relatively small test arena in our study, horses were not comfortable in the presence of cows. This study confirms the cows, as animate and therefore unpredictable objects, can provoke more avoidance than a mobile, yet inanimate, object. Heterospecific interactions and communication between these two species cannot be excluded as an explanation; this is an unexplored issue that deserves further investigation.

Additionally, our study, showing stronger cardiac activity during the hand-leading test in mares, indicates that sex differences in horses should be considered in temperament research and practical husbandry.

## Figures and Tables

**Table 1:** The variables measured in tests.

Measurement	Variable	Definition	Unit
Arena test	TimeSector	The total percentage of time the horse spent in Sector1–3 during the Control Stay or in the presence of Cow1, Cow2 or the Box	%
	Alert_occurr	The total number of occurrences of the following alert behaviours: vocalisations (whinny, neigh), snorts (loud, forceful burst of the air through the nostrils), defecations (eliminating faeces) during exposure to Control Stay, Cow1, Cow2 or Box	count
	Alert_dur	The total durations of the following alert behaviours: holding the tail high (above the horizontal line relative to its base) and the head and neck high (above the horizontal line relative to its withers) during exposure to Control Stay, Cow1, Cow2 or Box	seconds
Hand-leading test	HR	Mean heart rate during the 10 s stay at each stop	beats per minute (bpm)
	RMSSD	Mean RMSSD (Root mean square of successive differences between heartbeats) during the 10 s stay at each stop	milliseconds
	VASf	The researcher’s score of the perception of the fearfulness of the horse at stops 1–5. This was scored using a continuous scale from 0 (not at all afraid) to 100 (extremely fearful) by the researcher marking a point on a 100 mm length bar.	mm

**Table 2:** Statistical significance of Sector, Stimulus and the interaction of Sector and Stimulus (Sector * Stimulus) for TimeSector (the % of total time spent in each sector).

Variable	Sector	Stimulus	Sector * Stimulus	Sex	Age
	F; *p*				
TimeSector (%)	16,714.1; <0.01	5.18; <0.01	504.73; <0.01	0.18; 0.67	0.38; 0.53

**Table 3:** The time spent in Sectors 1, 2 and 3 as related to the stimulus.

Stimulus Variable	Control Stay	Cow1	Cow2	Box
	Mean, Standard Error			
TimeSector1 (%)	30.4, 0.24 AX	17.8, 0.20 BX	30.6, 0.24 AX	25.3, 0.22 CX
TimeSector2 (%)	20.7, 0.21 AY	24.6, 0.23 BY	19.8, 0.21 AY	22.7, 0.22 CY
TimeSector3 (%)	48.8, 0.26 AZ	58.2, 0.26 BZ	49.6, 0.26 AZ	52.1, 0.26 CZ

A, B, C: means with different letters in rows differ between stimuli at *p* < 0.01; X, Y, Z: means with different letters in columns differ between stimuli at *p* < 0.01.

**Table 4:** Statistical significance of Stop, Stimulus, the interaction of Stop and Stimulus (Stop * Stimulus), Sex and Age for HR, RMSSD and VASf.

Variable	Stop	Stimulus	Stop * Stimulus	Sex	Age
	F; *p*				
HR (bpm)	0.29; 0.89	133.0; <0.01	0.61; 0.77	5.27; 0.02	2.42; 0.12
RMSSD (ms)	1.44; 0.22	25.24; <0.01	0.90; 0.52	6.48; 0.01	0.52; 0.47
VASf (mm)	4.13; 0.01	7.78; <0.01	1.47; 0.17	0.07; 0.78	1.34; 0.25

**Table 5:** The effect of stimulus and sex on HR (bpm) and RMSSD (msec).

Variable	Stimulus			Sex	
	Control	Cow	Box	Geldings	Mares
	Mean 95% CI (Lower; Upper)				
HR	50.8 (45.2; 56.7) A	64.7 (67.7; 72.2) B	49.8 (44.4; 55.6) A	51.4 (43.3; 58.1) a	58.1 (43.0; 68.7) b
RMSSD	71.7 (52.3; 97.1) A	43.3 (31.8; 58.7) B	69.9 (51.3; 94.7) A	76.1(53.8; 107.0) A	47.4 (29.6; 75.6) B

A, B back-transformed means in rows for stimuli and sexes differ at *p* < 0.01; a, b back-transformed means in rows for stimuli and sexes differ at *p* < 0.05.

**Table 6:** Results from Spearman’s correlations between the experimenter’s rating of fearfulness (VASf) and physiological measures (HR–heart rate and RMSSD-root mean square of successive differences between heartbeats) at Stop1 and Stop5 during the hand-leading test.

		HR		RMSSD	
	Stimulus	*r_s_*	*p*	*r_s_*	*p*
	Control	0.31	0.17	−0.51	0.02
VASf,	Cow	0.48	0.03	−0.50	0.02
Stop1	Box	0.59	0.01	−0.41	0.07
	Control	0.04	0.85	−0.22	0.34
VASf,	Cow	0.30	0.20	−0.07	0.74
Stop5	Box	0.39	0.09	−0.44	0.05

## Data Availability

The data presented in this study are available on request from the corresponding author.

## Supplementary Materials

The following are available online at https://www.mdpi.com/2076-2615/11/11/3081/s1. Figure S1: Heart Rate (HR) in stops 1–5 when exposed to Box, Cow and in Control lead; Figure S2: Root Menu Square of Successive Differences between heartbeats (RMSSD) in stops 1–5 exposed to Box, Cow and in Control lead.
