# Correction: Wiśniewska et al. Heterospecific Fear and Avoidance Behaviour in Domestic Horses (*Equus caballus*). *Animals* 2021, *11*, 3081

**DOI:** 10.3390/ani12162026

**Published:** 2022-08-10

**Authors:** Anna Wiśniewska, Iwona Janczarek, Izabela Wilk, Ewelina Tkaczyk, Martyna Mierzicka, Christina R. Stanley, Aleksandra Górecka-Bruzda

**Affiliations:** 1 Department of Horse Breeding and Use, Faculty of Animal Sciences and Bioeconomy, University of Life Sciences in Lublin, 20-950 Lublin, Poland; 2 Animal Behaviour & Welfare Research Group, Department of Biological Sciences, University of Chester, Chester CH1 4BJ, UK; 3 Department of Animal Behaviour and Welfare, Institute of Genetics and Animal Biotechnology, Polish Academy of Sciences, 05-552 Magdalenka, Poland

The authors wish to make the following correction to this paper [1]:

In the original article, there was a mistake in the method used for heart rate analysis. The correct fragment appears below. The authors apologize for any inconvenience caused and state that the scientific conclusions are unaffected. The original article has been updated.

## Text Correction

On page 6, the last paragraph of 2.4. Hand-Leading Test:

The variables were analysed using Kubios HRV (version 2.1., Kuopio, Finland) software [22], as was described by Tarvainen et al. [23]. For artefact correction in RR (the interval between successive heart beats) analysis, the custom filter of the program was set at the maximal level.

should be revised to:

The variables were analysed using Polar ProTrainer5 software. No filter level was applied since clear, almost artefact-free RR curves were recorded (in three files the % of artefacts were 0.4%; 0.7%; 1.3%) [22,23].

## Reference Correction

22. Kubios HRV Software; version 2.1; Biomedical Signal Analysis Group, Department of Applied Physics, University of Kuopio: Kuopio, Finland, 2012.

should be revised to:

22. Von Borell, E.; Langbein, J.; Després, G.; Hansen, S.; Letterier, C.; Marchant-Forde, J.; Marchant-Forde, R.; Minero, M.; Mohr, E.; Prunier, A.; et al. Heart rate variability as a measure of autonomic regulation of cardiac activity for assessing stress and welfare in farm animals—A review. *Physiol. Behav.* **2007**, *92*, 293–316.

23. Tarvainen, M.P.; Niskanen, J.P.; Lipponen, J.A.; Ranta-Aho, P.O.; Karjalainen, P.A. Kubios HRV—Heart rate variability analysis software. *Comput. Methods Programs Biomed.* **2014**, *113*, 210–220, https://doi.org/10.1016/j.cmpb.2013.07.024.

should be revised to:

23. Malik, M. Heart rate variability: Standards of measurement, physiological interpretation, and clinical use: Task force of the European Society of Cardiology and the North American Society for Pacing and Electrophysiology. *Ann. Noninvasive Electrocardiol.* **1996**, *1*, 151–181, https://doi.org/10.1111/j.1542-474X.1996.tb00275.x.
