# Supplementary material for: Heterospecific Fear and Avoidance Behaviour in Domestic Horses (Equus caballus)
Source: Animals (Basel). 2021 Oct 28;11(11):3081. doi: 10.3390/ani11113081 (PMC8614530; doi:10.3390/ani11113081)
Supplement: Supplementary file 1 [file animals-11-03081-s001.zip › animals-1446657-supplementary.pdf]

Supplementary material

Heterospecific Fear and Avoidance Behaviour in Domestic Horses (*Equus caballus*)

Anna Wiśniewska [1], Iwona Janczarek [1], Izabela Wilk [1], Ewelina Tkaczyk [1], Martyna Mierzicka [1], Christina R. Stanley [2] and Aleksandra Górecka-Bruzda [3,*]

[1] Department of Horse Breeding and Use, Faculty of Animal Sciences and Bioeconomy, University of Life Sciences in Lublin, 20-950 Lublin, Poland; anna.wisniewska@up.lublin.pl (A.W.); iwona.janczarek@up.lublin.pl (I.J.); izabela.wilk@up.lublin.pl (I.W.); ewelina.tkaczyk@up.lublin.pl (E.T.); khiuk@up.lublin.pl (M.M.)

[2] Animal Behaviour & Welfare Research Group, Department of Biological Sciences, University of Chester, Parkgate Road, Chester CH1 4BJ, United Kingdom; christina.stanley@chester.ac.uk

[3] Department of Animal Behaviour and Welfare, Institute of Genetics and Animal Biotechnology, Polish Academy of Sciences, 05-552 Magdalenka, Poland

[*] Correspondence: a.gorecka@igbzpan.pl; Tel.: +48-22-736-71-24; Fax: +48-22-756-14-17

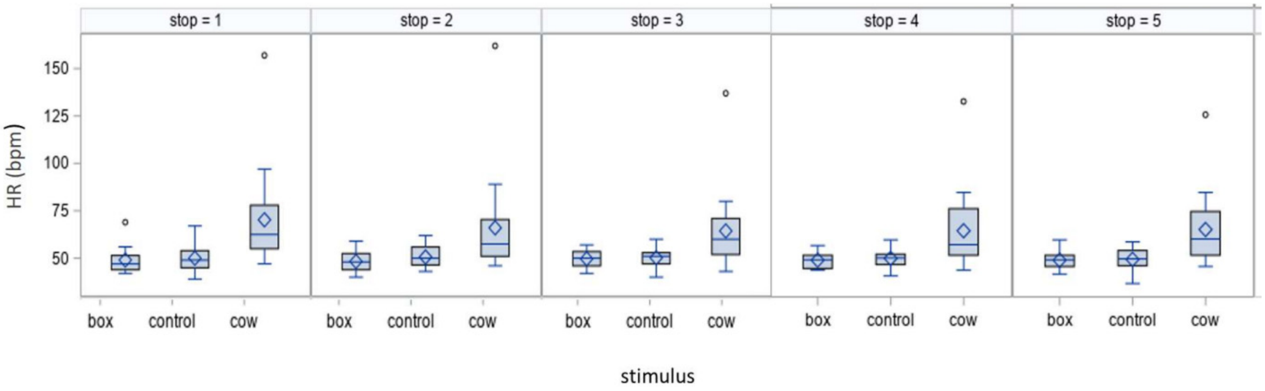

Figure S1. Heart Rate (HR) in stops 1–5 when exposed to Box, Cow and in Control lead.

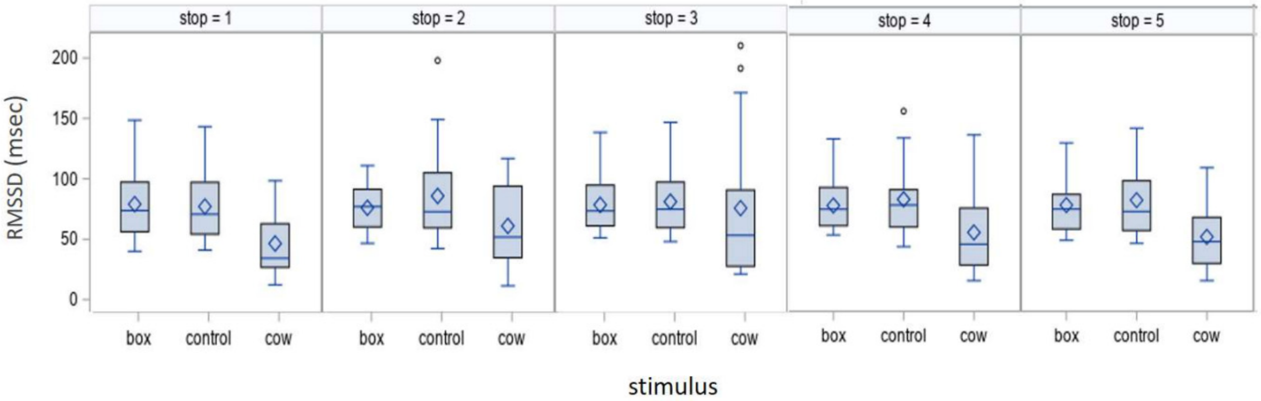

Figure S2. Root Menu Square of Successive Differences between heartbeats (RMSSD) in stops 1–5 exposed to Box, Cow and in Control lead.
